# Diagnostic value of ^18^F-fluorodeoxyglucose positron-emission tomography/computed tomography for preoperative lymph node metastasis of esophageal cancer: A meta-analysis

**DOI:** 10.1097/MD.0000000000013722

**Published:** 2018-12-14

**Authors:** Jingfeng Hu, Dengyan Zhu, Yang Yang

**Affiliations:** Department of Thoracic Surgery, The First Affiliated Hospital, Zhengzhou University, Zhengzhou, Henan 450052, China.

**Keywords:** esophageal cancer, lymph node metastasis, PET/CT

## Abstract

**Objective:**

We determined the value of ^18^F-fluorodeoxyglucose positron-emission tomography/computed tomography (^18^FDG PET/CT) for the assessment of preoperative lymph node metastases in patients with esophageal cancer.

**Methods:**

We searched electronic database indexes for articles on PET/CT assessment of lymph node status. Information including true positives, false positives, false negatives, and true negatives was obtained. Based on these data, the pooled sensitivity, specificity, diagnostic odds ratio, and likelihood ratio were calculated using bivariate models and receiver operating characteristic curves (ROCs) were drawn.

**Results:**

Patients without neoadjuvant treatment had a pooled sensitivity and specificity (95% confidence interval [CI]) of 0.57 (0.45–0.69) and 0.91 (0.85–0.95), respectively. Patients who received neoadjuvant treatment had a pooled sensitivity and specificity of 0.53 (0.35–0.70) and 0.96 (0.86–0.99), respectively.

**Conclusions:**

The PET/CT has a high diagnostic specificity but its diagnostic sensitivity is low; thus, its diagnosis findings cannot accurately reflect the lymph node status.

## 1 Introduction

Esophageal cancer (EC) is a highly malignant disease with a very poor prognosis. Lymph node metastasis in patients with EC is an important prognostic factor.^[1]^ Patients with stage N2 disease and above have a decreased survival rate.^[2]^ The 2- and 5-year recurrence rates of EC are highly correlated with lymph node status.^[3]^ Extensive lymph node dissection may provide better healing but the technical requirements are high and the potential risk of postoperative complications is dramatically increased.^[4,5]^ In China, the proportion of elderly people with EC is increasing and many have poorer cardiopulmonary function. Therefore, less harmful treatments may provide better recovery following surgery. The accurate assessment of lymph node metastasis in patients with EC is complicated but still important for predicting disease progression, choice of treatment plan, preoperative staging, etc.^[6,7]^ When selective lymph node dissection is performed, the preoperative tools for the precise positioning of lymph node metastasis become very important in order to determine a clear range.^[4,8]^

Currently, the techniques for detecting lymph node metastasis in patients with EC include computed tomography (CT), ultrasonography (US), endoscopic ultrasonography (EUS), and positron-emission computed tomography (PET-CT). The uptake of ^18^FDG, a radiopharmaceutical for PET-CT, is a marker of glucose uptake by tissues which is closely related to tissue metabolism. Therefore, PET-CT is used to evaluate lymph node metastasis in EC.^[9,10]^ A maximum standardized uptake value (SUV_max_) in PET-CT greater than 2.5 and a tumor diameter > 1 cm are considered to be indicative of a malignant tumor.^[11,12]^

Although a number of studies have used ^18^FDG PET-CT to asses the status of preoperative lymph nodes in patients with EC, the results are not always in agreement; thus, the effects of treatment remain controversial. Results and guidelines regarding the use of PET-CT for the diagnosis of esophageal neoplasms and regional lymph node metastasis in patients with EC are lacking.^[11,13,14]^ The continuous advancement of science and technology including those in imaging equipment and control software has led to the development of PET-CT technology and equipment that can provide more accurate tumor diagnosis and staging. The present meta-analysis incorporated and analyzed the latest data using PET/CT technology during the past 5 years. The aim was to determine the significance of implementing PET/CT, particularly for the evaluation of lymph node metastasis in EC.

## 2 Methods

All analyses were based on previous published studies, thus no ethical approval and patient consent are required.

## 3 Search Strategy

A comprehensive search was conducted of the MEDLINE and PubMed electronic databases for articles published between January 1, 2013 and December 31, 2017. The search strategy comprised a combination of the following terms: esophageal cancer or esophageal carcinoma; lymph node or lymph node metastasis or lymph node staging.; and positron emission tomography or PET/CT. The search was limited to studies published in English. The reference lists were manually filtered to identify additional related articles.

### 3.1 Study selection and quality evaluation

The relevant data were independently extracted by 2 reviewers. The data were recorded on a standardized form, and disagreements were resolved by all authors. The inclusion criteria were as follows: patient pathology confirmed to be EC; lymph node status detected by PET/CT before surgery, for patients treated with neoadjuvant therapy, lymph node status was detected by PET/CT after neoadjuvant therapy and before surgery; use of fluorodeoxyglucose as the PET/CT tracer; histopathological results of lymph node assessment followed gold standards; contained complete information including true positives, false positives, false negatives, and true negatives that could be constructed into a complete 4-squared table; included at least 10 patients.

The exclusion criteria included studies in which the patients who received preoperative neoadjuvant treatment could not be accurately distinguished.

Quality Assessment of Diagnostic AccuracyStudies-2 (QUADAS-2) was used to evaluate the quality of the studies.

### 3.2 Data analysis

The descriptive data of the literature were extracted in tabular form, including the author, date of publication, country, number of cases, gender, etc. Heterogeneity among these studies was assessed by Q-tests. Data including true positives, false positives, false negatives, and true negatives were extracted from each study and used to generate receiver operating characteristic curves (ROCs). Bivariate regression models were used for the meta-analysis. All data analysis was performed using Stata 12.0.

## 4 Results

### 4.1 Search Results

A total of 105 documents were retrieved, 88 of which were excluded after reading the titles and abstracts; an additional three articles were rejected according to the exclusion criteria. Therefore, a total of 14 articles were included in this meta-analysis (Table 1).^[6,7,11,15–25]^

**Table 1:**
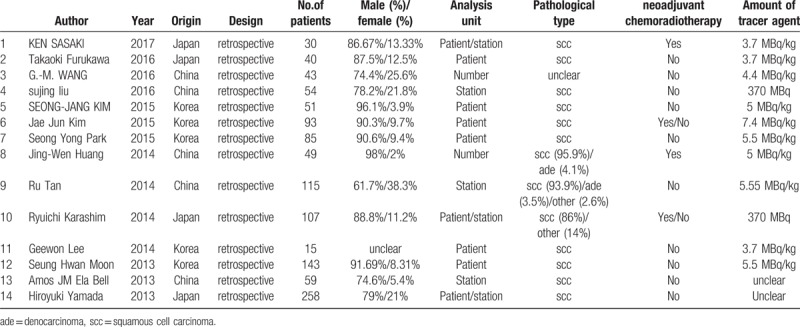
The clinical characteristics.

### 4.2 Study description

**Figure 1:**
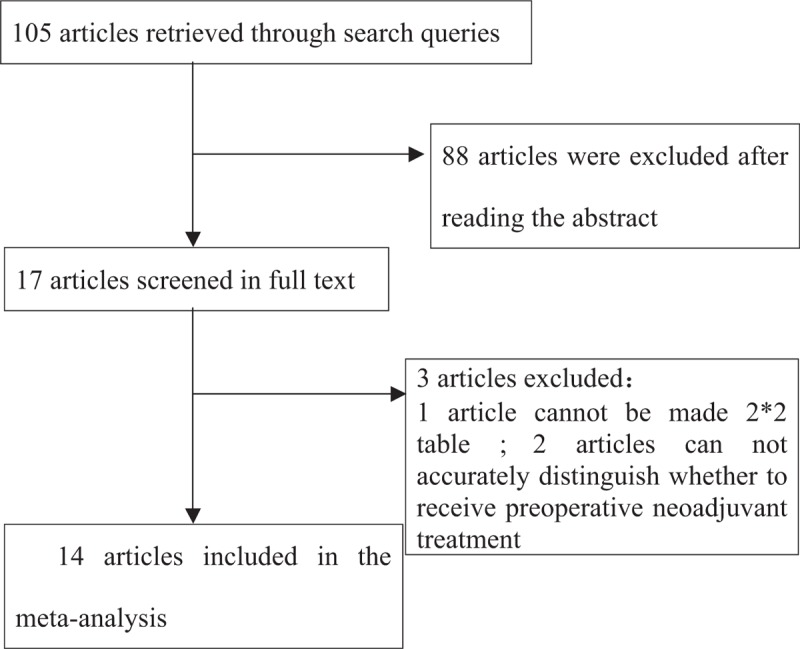
The flow chart of studies retrieval.

The basic information of the 14 studies included in the meta-analysis is shown in Figure 1. A 9, 6, and 2 studies utilized per-patient, per-station analysis, and per-number analyses, respectively. Pathological types of esophageal squamous cell carcinoma occurred in 13 studies, esophageal adenocarcinomas in 2 studies, and 1 study did not clearly indicate. Twelve studies did not perform preoperative neoadjuvant treatment, while 4 studies did.

### 4.3 Literature quality evaluation

All included studies were retrospective. Among the 11 questions in QUADAS-2, a quality assessment tool for diagnostic accuracy studies, 3 studies responded 11 “yes,” 9 studies responded 10 “yes,” and 2 studies responded 9 “yes.”

### 4.4 Diagnostic accuracy of PET/CT

**Figure 2:**
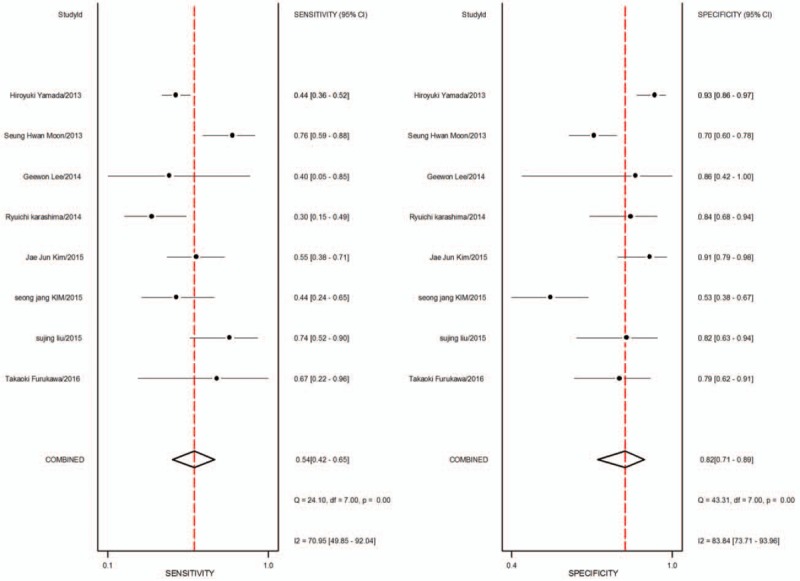
The forest map of sensitivity and specificity for preoperative neoadjuvant therapy was not performed and results analysis was based on per-patient.

**Figure 3:**
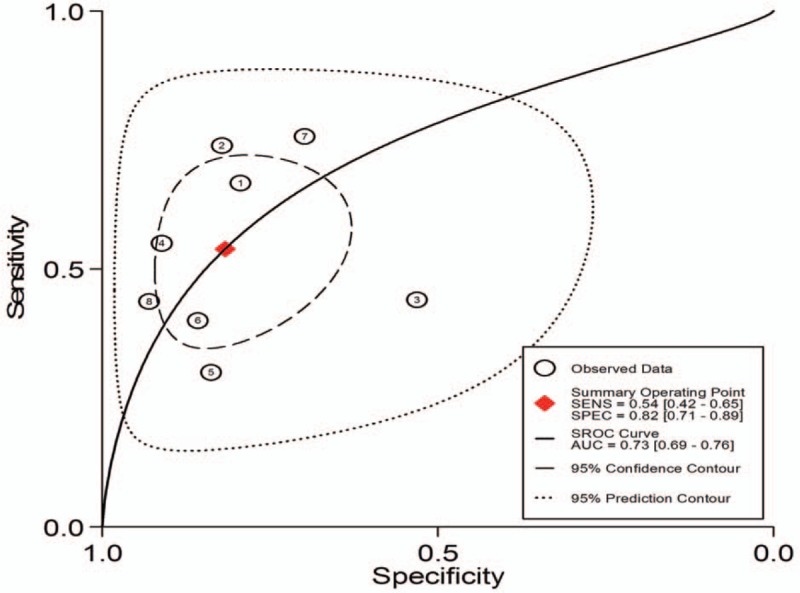
The SROC with prediction and confidence contours for preoperative neoadjuvant therapy was not performed and results analysis was based on per-patient. ROC = receiver operating characteristic curves.

**Figure 4:**
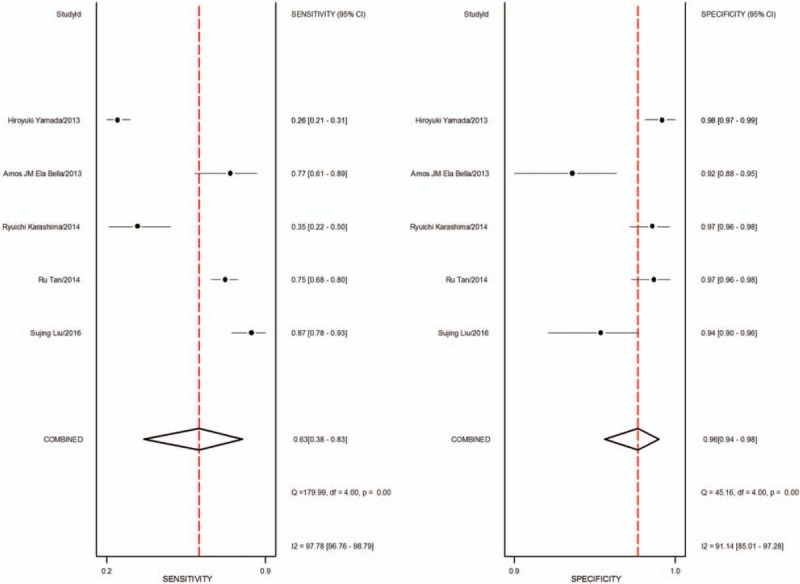
The forest map of sensitivity and specificity for preoperative neoadjuvant therapy was not performed and results analysis was based on per-station.

**Figure 5:**
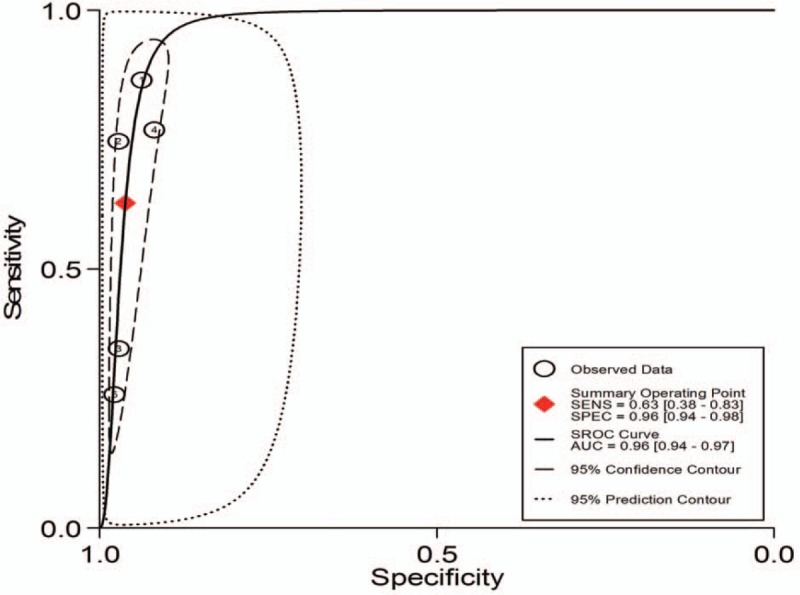
The SROC with prediction and confidence contours for preoperative neoadjuvant therapy was not performed and results analysis was based on per-station. ROC = receiver operating characteristic curves.

**Figure 6:**
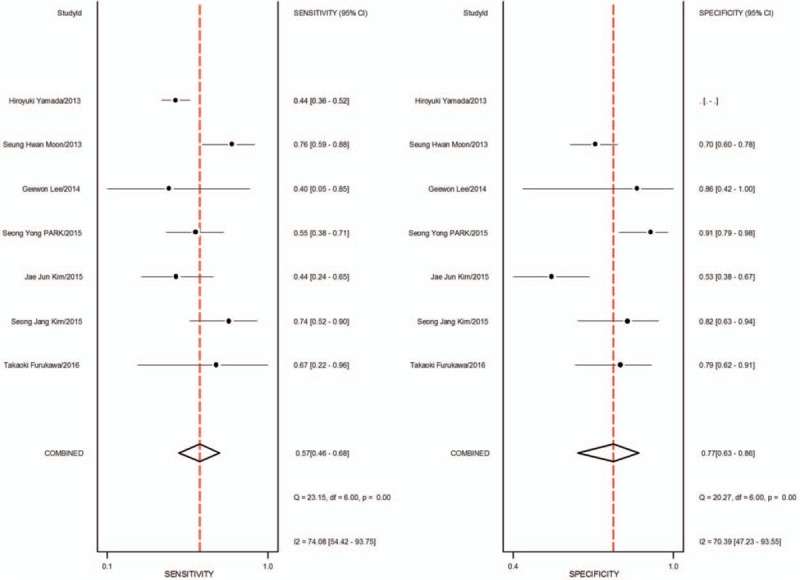
The forest map of sensitivity and specificity for preoperative neoadjuvant therapy was not performed and results analysis was based on per-patient (esophageal squamous cell carcinoma).

**Figure 7:**
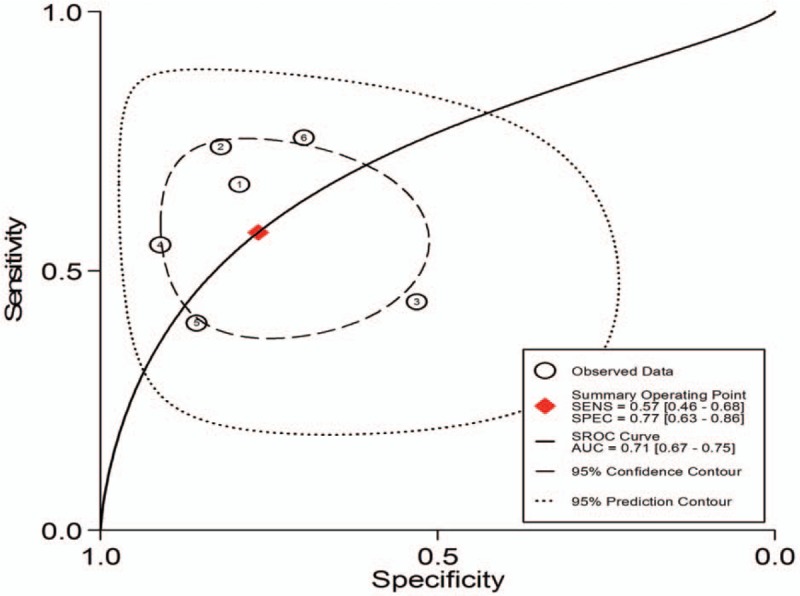
The SROC with prediction and confidence contours for preoperative neoadjuvant therapy was not performed and results analysis was based on per-patient (esophageal squamous cell carcinoma). ROC = receiver operating characteristic curves.

**Figure 8:**
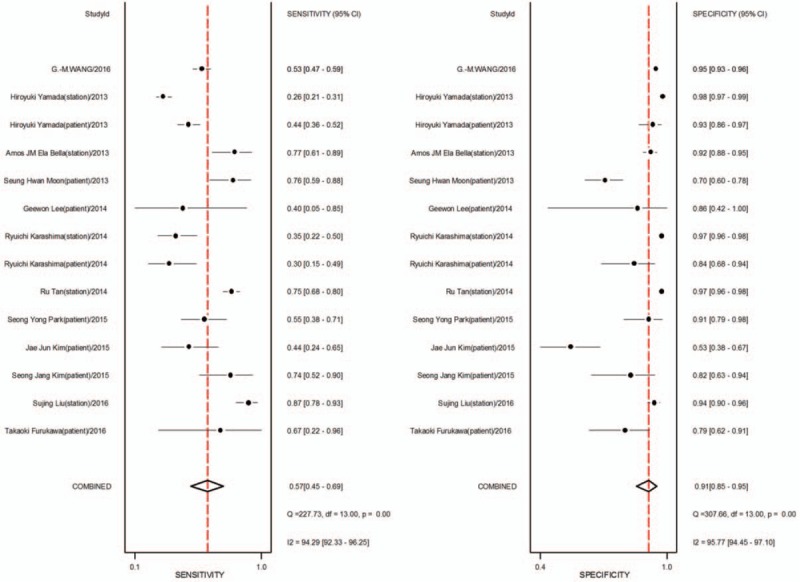
The forest map of sensitivity and specificity for preoperative neoadjuvant therapy was not performed and results analysis was based on per-patient, per-station and per-number.

**Figure 9:**
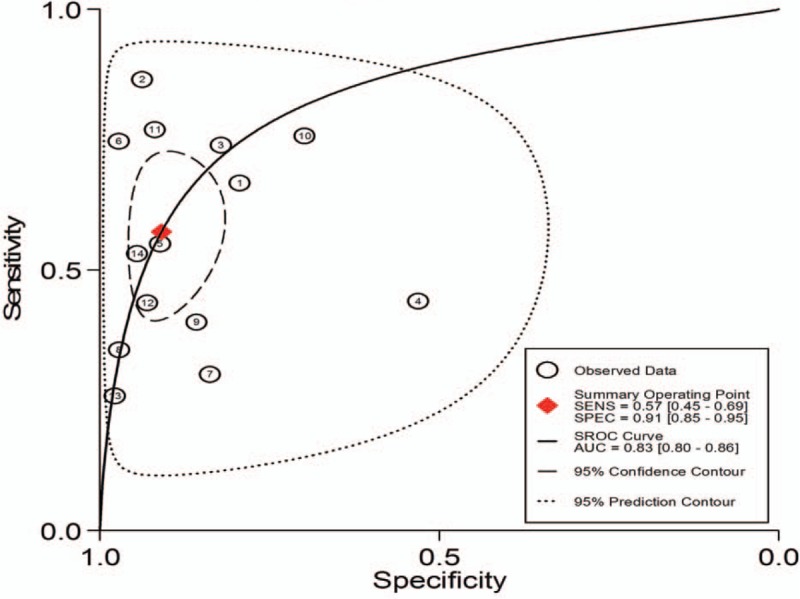
The SROC with prediction and confidence contours for preoperative neoadjuvant therapy was not performed and results analysis was based on per-patient, per-station and per-number ROC = receiver operating characteristic curves.

**Figure 10:**
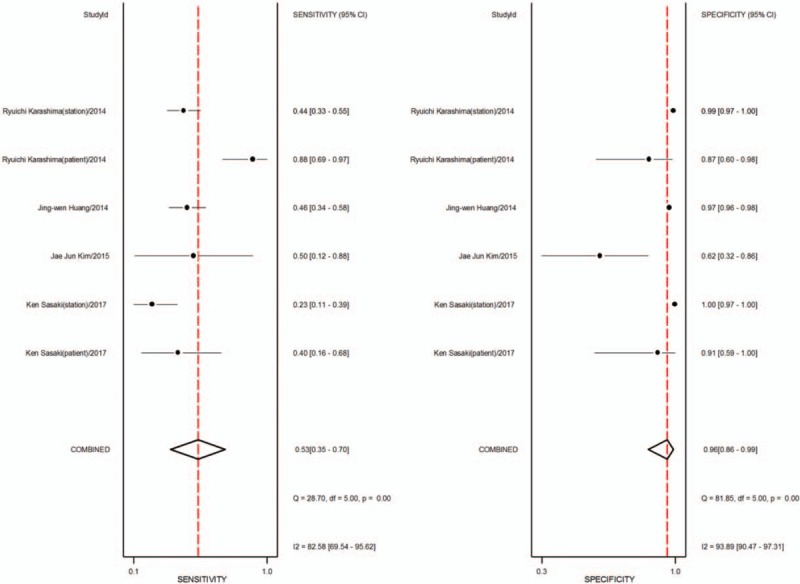
The forest map of sensitivity and specificity for preoperative neoadjuvant therapy was performed and results analysis was based on per-patient, per-station and per-number.

**Figure 11:**
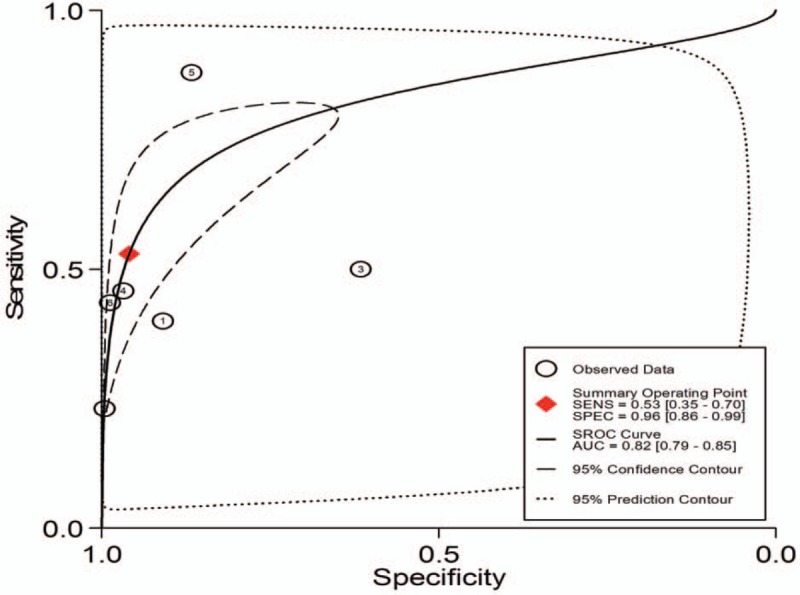
The SROC with prediction and confidence contours for preoperative neoadjuvant therapy was performed and results analysis was based on per-patient, per-station and per-number. ROC = receiver operating characteristic curves.

1. Preoperative neoadjuvant therapy was not performed, with results based on per-patient analysis. Data extracted from 8 studies were used to draw forest maps, as shown in Figure 2. The Q-test *P* < .01 indicated heterogeneity between studies. For these 8 studies, the combined pooled sensitivity, specificity, positive likelihood ratio, negative likelihood ratio, and diagnostic ratios (95% confidence interval [CI]) were 0.54 (0.42–0.65), 0.82 (0.71–0.89), 2.9 (1.8–4.8), 0.56 (0.43–0.73), and 5 (3–10), respectively. Figure 3 shows the ROC curves. The area under the curve was 0.73 (95% CI, 0.69–0.76).
2. Preoperative neoadjuvant therapy was not performed, with results based on per-station analysis. Data extracted from 5 studies were used to draw forest maps, as shown in Figure 4. The Q-test *P* < .01 indicated heterogeneity between studies. For these 5 studies, the combined pooled sensitivity, specificity, positive likelihood ratio, negative likelihood ratio, and diagnostic ratio (95% CI) were 0.63 (0.38–0.83), 0.96 (0.94–0.98), 16.4 (12.1–22.3), 0.39 (0.21–0.73), and 42 (20–90), respectively. Figure 5 shows the ROC curves. The area under the curve was 0.96 (95% CI, 0.94–0.97).
3. Preoperative neoadjuvant therapy was not performed, with results based on per-patient analysis (esophageal squamous cell carcinoma). Data extracted from 7 studies were used to draw forest maps, as shown in Figure 6. The Q-test *P* < .01 indicated heterogeneity between studies. For these 7 studies, the combined pooled sensitivity, specificity, positive likelihood ratio, negative likelihood ratio, and diagnostic ratio (95% CI) were 0.57 (0.46–0.68), 0.77 (0.63–0.86), 2.5 (1.4–4.3), 0.56 (0.40–0.77), and 4 (2.10), respectively. Figure 7 shows the ROC curves. The area under the curve was 0.71 (95% CI, 0.67–0.75).
4. Preoperative neoadjuvant therapy was not performed, with per-patient, per-station, and per-number analyses. Data extracted from 12 studies was used to draw forest maps, as shown in Figure 8. The Q-test *P* < .01 indicated heterogeneity between studies. For these 12 studies, the combined pooled sensitivity, specificity, positive likelihood ratio, negative likelihood ratio, and diagnostic ratio (95% CI) were 0.57 (0.45–0.69), 0.91 (0.85–0.95), 6.3 (3.7–10.8), 0.47 (0.36–0.62), and 13 (7–27), respectively. Figure 9 shows the ROC curves. The area under the curve was 0.83 (95% CI, 0.80–0.86).
5. Preoperative neoadjuvant therapy was performed, with per-patient, per-station, and per-number analyses. Data extracted from four studies were used to draw forest maps, as shown in Figure 10. The Q-test *P* < .01 indicated heterogeneity between studies. For these 4 studies, the combined pooled sensitivity, specificity, positive likelihood ratio, negative likelihood ratio, and diagnostic ratio (95% CI) were 0.53 (0.35–0.70), 0.96 (0.86–0.99), 13.0 (4.8–34.8), 0.49 (0.35–0.69), and 26 (12–57), respectively. Figure 11 shows the ROC curves. The area under the curve was 0.82 (95% CI 0.79–0.85).

## 5 Discussion

Lymph node metastasis is an important prognostic factor in EC.^[26]^ Studies have shown that N staging and the numbers of distant lymph node metastasis have important prognostic implications in patients with EC and are reliable prognostic factors for surgical treatment.^[24,27,28]^ Therefore, the lymph node status has important significance for guiding treatment. At present, PET/CT that contains more detailed anatomical information and tissue metabolic information than traditional methods of examination has become essential for the assessment of lymph node metastasis in patients with EC.^[20,24]^

Sensitivity and specificity are 2 basic features of a diagnostic experiment. Sensitivity refers to the proportion of positive cases detected by diagnostic tests in the group of cases diagnosed using standard diagnostic methods, in which a higher sensitivity indicates a lower rate of missed diagnosis. Specificity refers to the proportion of negative results detected by diagnostic tests in a control group diagnosed as disease-free by standard diagnostic methods, in which a higher specificity indicates a lower rate of misdiagnosis. In this study, PET/CT was less sensitive to preoperative evaluation of lymph node metastases. In patients without preoperative neoadjuvant treatment, the pooled sensitivity (95% CI) in per-patient and per-station analyses were 0.54 (0.42–0.65) and 0.63 (0.38–0.83), respectively. In patients with esophageal squamous cell carcinoma, the pooled sensitivity in per-patient analysis was 0.57 (0.46–0.68). This difference may be related to micrometastasis in distant lymph nodes. Studies have shown that most distal metastatic lymph nodes have diameters < 6 mm, while the PET/CT diagnostic criteria are ≥ 1 cm in diameter, thus resulting in a lower diagnostic sensitivity.^[29]^

The positive and negative likelihood ratios are more clinically significant compared to sensitivity and specificity. The likelihood ratio reflects the credibility of the diagnosis: the higher the positive likelihood ratio, the more likely it is to be a true positive finding when the test result is positive; similarly, the lower the negative likelihood ratio, the more likely it is to be a true negative finding when the test result is negative. It is generally accepted clinically that a positive likelihood ratio greater than 10 or a negative likelihood ratio less than 0.1 indicate a significantly increased possibility of diagnosis or exclusion. In this study, regardless of pathological type or if limited to squamous cell carcinoma patients, without preoperative neoadjuvant treatment, the positive likelihood ratios based on per-patient analysis were lower, at 2.9 (1.8, 4.8) and 2.5 (1.4,4.3), respectively. Per-station analysis resulted in a positive likelihood ratio of 16.4 (12.1, 22.3) and a negative likelihood ratio of only 0.39 (0.21, 0.73). These data indicate that PET/CT is less accurate for assessing lymph node metastases.

PET/CT has an advantage as an indicator of biological activity, which is conducive to the detection of lymph node status, especially after preoperative treatment. Surgical treatment after neoadjuvant radiotherapy improves survival rates is considered the standard treatment for patients with locally-advanced esophageal squamous cell carcinoma.^[15]^ Neoadjuvant therapy increases the resectability, overall survival, and disease-free survival rates in patients with EC.^[30]^ For locally-advanced EC, a meta-analysis comparing esophagectomy after preoperative neoadjuvant therapy to surgery alone showed beneficial effects on the disease-free survival rate and local area control.^[31]^ In addition, patients who did not respond to preoperative neoadjuvant therapy had a lower 5-year survival rate compared to that of patients with a good preoperative neoadjuvant response (downstage) (0–10.7% vs. 34.9–53%).^[20]^ Adham et al reported improved survival rates in patients who responded well to preoperative neoadjuvant therapy followed by radical surgery, while patients with adverse reactions had a poor prognosis after radical surgery. The prognosis of these patients was not related to the tumor stage before treatment but rather was closely related to whether the neoadjuvant therapy was in decline.^[30]^ The survival rates of good responders are improved by surgery. Therefore, in order to better formulate a treatment strategy, it is necessary to evaluate the responses before surgery and after neoadjuvant therapy. The status of pathological LN metastasis has been shown to be the strongest predictor of survival in patients with EC after neoadjuvant therapy.

Therefore, the prediction of the prognosis in patients with EC should consider the state of pathological LN metastases rather than residual primary tumors.^[15]^ Additionally, because the size of the lymph nodes after neoadjuvant therapy is not always related to the treatment response, PET/CT as an indicator of biological activity is conducive to the detection of lymph node status, especially after preoperative treatment.^[22]^ The present study analyzed the accuracy of PET/CT evaluation of lymph node metastasis in EC following neoadjuvant therapy, showing pooled sensitivity, specificity, positive likelihood ratio, negative likelihood ratio, and diagnostic ratios (95% CI) of 0.53 (0.35–0.70), 0.96 (0.86–0.99), 13.0 (4.8–34.8), and 0.49 (0.35–0.69), respectively. These values are similar to those of patients undergoing direct surgery, suggesting that neoadjuvant therapy has little effect on the ability of PET/CT to predict lymph node status.

The present study also showed that PET-CT has high specificity but a relatively low sensitivity for the detection of lymph node status. This finding allows doctors to better understand and implement PET-CT in clinical practice. The high specificity of PET-CT for the detection of lymph node status can be used to confirm that lymph nodes are not metastasized. Surgery for EC is generally performed in 3 fields but is not suitable for every patient. For example, although the importance of para-recurrent laryngeal nerve lymphadenectomy (RLNL) has gradually become a consensus,^[32]^ it can cause serious complications such as hoarseness and choking after drinking water, which can seriously affect postoperative recovery and may be life-threatening. In these particular circumstances, PET-CT can be used to exclude patients without lymph nodes metastasis for lymph node dissection during radical surgery for EC, thereby reducing the scope of surgical cleaning, shortening the operation time, and reducing the harm of surgery.

The limitation of our meta-analysis was there were relatively few studies on the accuracy of PET/CT evaluation of lymph node metastasis of EC after neoadjuvant therapy compared to the number of studies that did not include neoadjuvant therapy, which may have affected the comparisons between these studies.

## 6 Conclusion

In summary, PET/CT has a high diagnostic specificity but a low diagnostic sensitivity; thus, the diagnosis results cannot accurately reflect the lymph node status. Although accurate N staging is not possible, PET/CT has good test specificity and can be used to rule out lymph node metastasis and narrow the scope of cleansing.

## Author contributions

**Data curation:** Jingfeng Hu, Dengyan Zhu.

**Investigation:** Jingfeng Hu, Dengyan Zhu.

**Writing – original draft:** Jingfeng Hu.

**Writing – review & editing:** Yang Yang.
